# Pre-operative language ability in patients with presumed low-grade glioma

**DOI:** 10.1007/s11060-017-2699-y

**Published:** 2017-12-01

**Authors:** Malin Antonsson, Francesca Longoni, Asgeir Jakola, Magnus Tisell, Magnus Thordstein, Lena Hartelius

**Affiliations:** 1 Institute of Neuroscience and Physiology, Speech and Language Pathology Unit, Sahlgrenska Academy at the University of Gothenburg, Box 452, 405 30 Gothenburg, Sweden; 2 Department of Neurosurgery, Sahlgrenska University Hospital, 413 45 Gothenburg, Sweden; 3 Institute of Neuroscience and Physiology, Sahlgrenska Academy at the University of Gothenburg, Gothenburg, Sweden

**Keywords:** Low-grade glioma, Brain tumor, Language tests, Aphasia

## Abstract

In patients with low-grade glioma (LGG), language deficits are usually only found and investigated after surgery. Deficits may be present before surgery but to date, studies have yielded varying results regarding the extent of this problem and in what language domains deficits may occur. This study therefore aims to explore the language ability of patients who have recently received a presumptive diagnosis of low-grade glioma, and also to see whether they reported any changes in their language ability before receiving treatment. Twenty-three patients were tested using a comprehensive test battery that consisted of standard aphasia tests and tests of lexical retrieval and high-level language functions. The patients were also asked whether they had noticed any change in their use of language or ability to communicate. The test scores were compared to a matched reference group and to clinical norms. The presumed LGG group performed significantly worse than the reference group on two tests of lexical retrieval. Since five patients after surgery were discovered to have a high-grade glioma, a separate analysis excluding them were performed. These analyses revealed comparable results; however one test of word fluency was no longer significant. Individually, the majority exhibited normal or nearly normal language ability and only a few reported subjective changes in language or ability to communicate. This study shows that patients who have been diagnosed with LGG generally show mild or no language deficits on either objective or subjective assessment.

**Electronic supplementary material:**

The online version of this article (10.1007/s11060-017-2699-y) contains supplementary material, which is available to authorized users.

## Introduction

Low-grade gliomas (LGG) are slow-growing brain tumors that are often located near or within so-called eloquent areas involving language, motor, or sensory functions [1]. Surgery to remove them therefore carries a risk of leaving patients with problems such as aphasia and other cognitive impairments. However, the tumor itself may also displace or infiltrate language areas of the brain and give rise to deficits [2].

Language impairment in LGG patients has been studied largely in patients with tumors in or near language eloquent areas of the brain and who have undergone awake surgery (e.g. [3–9]). The prevalence of language impairment prior to surgery in patients who have a tumor in the language areas in the left brain hemisphere (LH) has been reported to be between 10.4 and 36.4% [4–6, 10]. The differences in reported prevalence may be due to differences in methods of selecting patients as well as in assessing them. Many studies include patients with various grades of tumor, patients with recurrent tumors and/or patients who have already undergone treatment, and all of these factors may cause language impairment.

There is some research showing that patients who have a tumor outside of the classical language areas of both hemispheres may also present with language impairment [11–13]. This is supported by findings from intraoperative language mapping, which show that in cases of glioma, there may be heterogeneous localization of language areas [6, 14].

Common language problems found in LGG patients are word retrieval deficits, which can be seen in naming or word fluency tasks (e.g. [8, 9, 15]). Word retrieval difficulties are also a commonly self-reported deficit [8, 16].

Several studies stress the importance of using assessment methods in these patients that are sensitive enough to enable detection of subtle deficits [4, 8]. Nevertheless, many studies use standard aphasia test batteries that are designed to assess deficits resulting from stroke (e.g. [5, 10–12]).

There are few studies of language impairment in newly diagnosed, untreated LGG patients. Satoer et al. [17] investigated spontaneous speech by analysing various linguistic variables in 27 glioma patients, including patients with LGG, before and after surgery. These patients had a higher frequency of incomplete sentences and problems with naming and word fluency than a matched control group. Difficulties in naming and/or word fluency have also been found in studies focusing on cognitive function in newly diagnosed LGG patients [15, 16], but these studies have been constrained to testing limited language abilities. Whether or not other language deficits occur in newly diagnosed LGG patients has not been sufficiently investigated. One aspect of language not previously investigated is high-level language i.e. more complex language abilities demanding extensive language and cognitive processing [18]. HLL difficulties have previously been found in patients with different neurological conditions including multiple sclerosis, and Parkinson´s disease [19]. The value of these measures is that they are sensitive to subtle language/cognitive disabilities that are not captured by traditional language tests.

Our study aimed to investigate language ability in newly diagnosed patients with presumed LGG using a highly sensitive and comprehensive language test battery, including tests of high-level language. We compared the patients’ performance with a matched reference group. We also investigated whether the occurrence of language impairment differed depending on location in language eloquent area, and whether the patients reported any subjective change in language, speech or communicative ability.

## Method

### Participants

Consecutive patients with presumed LGG who presented at the neurosurgical department of Sahlgrenska University hospital in Gothenburg between November 2014 and September 2016 were asked to participate. Diagnosis had been based on MRI scans, physical examination and history.

Patients whose mother tongue was not Swedish were excluded (2 patients), as were those who had previously undergone brain surgery and/or other tumor treatments (8 patients) or who had moderate or severe developmental language or cognitive disorders (1 patient). A total of thirty adult patients were asked to participate and 25 patients agreed to. Two of these were found not to be suffering from glioma (one had a meningioma and one, inflammation) and they were therefore excluded, leaving 23 patients in the group. Two of those included (patients number 2 and 19) had difficulty spelling but no other developmental language disorders. Histological examination showed that 18 patients had a LGG (grade II), and five had a tumor of grade III or IV. Since the criterion for inclusion was to have been diagnosed with a *presumed* LGG, all 23 patients are included in the analyses (described in detail in Table 1.) However, an additonal analysis was made, excluding the patients with a glioma of grade III and IV.

**Table 1:** Demographic and clinical characteristics of the included patients N = 23

ID	Sex/age/education (y)	Handedness	Tumor characteristics					Seizures
			Laterality	Location	Near eloquent area^a^	Histology	Volume T2/Flair ml	
P4	M/60/13	R	Left	Temporal, insula	Yes (language, motor)	OA II	22.3	No
P12	M/31/22	R	Left	Frontal	Yes (language, motor)	A IV (GBM)	45.1	Yes
P14	M/52/16	R	Left	Frontal, insula	Yes (language)	A IV (GBM)	39.7	Yes
P15	M/46/15	R	Left	Multifocal	Yes (several)	A II	65.6	Yes
P25	M/25/12	R	Left	Fronto-temporal	Yes (language)	O III	27.2	Yes
P32	M/53/13	R	Left	Frontal	Yes (language, motor)	O II	87.8	Yes
P36	M/24/12	L	Left	Frontal	Yes (language)	A II	7.8	No
P1	M/45/11	R	Left	Frontal	Yes (motor)	OA III	80.0	Yes
P2	F/37/19	R	Left	Temporal	No	A II	10.6	No
P5	F/57/16	R	Left	Frontal	Yes (motor)	OA II	63.2	Yes
P18	F/49/12	R	Left	Parietal	No	Ganglioglioma I	2.8	No
P19	M/26/20	R	Left	Temporal	No	A II	12.1	Yes
P21	F/64/12	R	Left	Temporal	No	A II	5.5	No
P27	M/56/15	R	Left	Temporal	No	A II	50.8	Yes
P29	M/26/16	R	Left	Temporal	No	Ganglioglioma II	4.2	Yes
P34	M/67/17	R	Left	Frontal	No	O II	8.7	No
P7	M/43/20	R	Right	Gyrus cingula	No	OA III	7.0	Yes
P10	F/42/11	R	Right	Parietal	No	OA II	7.7	No
P11	F/56/12	R	Right	Insula, frontal	Yes (motor)	O II	68.8	Yes
P13	F/39/16	R	Right	Frontal	No	O II	83.0	Yes
P20	F/42/12	R	Right	Frontal	Yes (motor)	A II	1.4	Yes
P26	M/44/17	R	Right	Frontal, temporal insula, thalamus	Yes (motor)	O II	150.3	Yes
P35	M/43/11	L	Right	Frontal	No	Ganglioglioma II	9.2	Yes

*F* female, *M* male, *R* right, *L* left, *OA* Oligoastrocytoma, *A* Astrocytoma, *O* Oligodendro-glioma

^a^Eloquence defined according to Chang et al. [20], further described in the section on data collection

The study also included a reference group (R-group) consisting of adults who had no known neurological disease (*N* = 80). These people were selected from a larger group, described by Antonsson et al. [23]. The groups were matched at group level with respect to age (Age: presumed LGG-group *median* 44 years, *IQR* 19, Reference-group *median* 46 years, *IQR* = 23, *U* = 869.5, *p* = .689) and educational level (presumed LGG-group *median* 15 years, *IQR* = 5, Reference group *median* 15 years, *IQR* = 4; *U* = 843.5, *p* = .542). In the additional analyses on the 18 patients with LGG the same reference group was used for comparisons since no difference were found between the groups with respect to age (LGG-group *median* 45 years, *IQR* 18, Reference-group *median* 46 years, *IQR* = 23, *U* = 711.5, *p* = .938) or educational level (LGG-group *median* 13 years, *IQR* = 4.3, Reference group *median* 15 years, *IQR* = 4; *U* = 584, *p* = .209).

### Language assessment

The test battery (Table 2) included Swedish standard tests to detect aphasia, and instruments for detecting more subtle language disorders. The latter included tests of lexical retrieval (naming and word fluency) and a test (BeSS) [22] of high-level language functions (HLL).

**Table 2:** A description of the language task in the test battery

LGG	RG	Tests (max score)	Description
x	–	A-ning (220) [21]	A Swedish aphasia test that assesses informative speech, repetition, auditory comprehension, written comprehension, dictation, and written information. Gives a profile of the aphasia symptoms and their severity
x	x	BeSS (210) [22]	A test of high-level language. Demands a higher level of production and comprehension. It consists of seven subtests, see description for each subtest. Scoring and norms according to Antonsson et al. [23]
		Subtests in BeSS (30)	
x	x	1. Repetition of long sentences	Repeat sentences 9–16 words in length. The sentences consist of main clauses and subordinate clauses
x	x	2. Recreating sentences	Create a syntactically, semantically and pragmatically adequate utterance using three given words and a given context
x	x	3. Making inferences	Listen and read a text and answer questions about issues not explicitly stated in the text
x	x	4. Comprehension of logico-grammatical sentences	Answer questions or follow instructions consisting of sentences with complex grammatical structures, such as double negations, inverted sentences and multi-step instructions
x	x	5. Comprehension of ambiguous sentences	Give two different interpretations of sentences containing lexical or syntactic ambiguities
x	x	6. Comprehension of metaphors	Explain the meaning of sentences containing metaphorical expressions
x	x	7. Word definitions	Define or offer a synonym for various words
x	x	Sentence analysis (54) [24]	A morphological test in which the subject is asked to listen to sentences, and then repeat them, counting the number of words each sentence contains. Scoring follows Elbro [24] and results are compared to unpublished norms
x	x	Morphological completion (45) [24]	A morphological test in which the subject is asked to complete a word that is missing a morpheme at the beginning or end. Scoring follows [24] and results are compared to unpublished norms
x	x	Boston naming test (BNT) (60) [25]	A test of confrontation naming. The subject is asked to name pictures of nouns. In the present study, BNT is presented on a computer (digitalization of picture material with the permission of the copyright owner: Kaplan, Goodglass & Weintraub, [26]). Scoring and norms according to Tallberg et al. [27]
x	x	Word fluency [28]	Word fluency measures a person’s ability to generate words in a particular category within a limited time. The present study includes both letter and semantic fluency. Administration and norms according to Tallberg et al. [29]
		FAS (letter fluency)	Generate as many words as possible that begin with F, A or S in 1 min
		Animals (semantic fluency)	Generate as many words as possible that belong to the category animals in 1 min
		Verbs (semantic fluency)	Generate as many verbs as possible in 1 min
x	–	Token test (36) [30] version C, Swedish standardization [31]	A test of auditory comprehension. The subject is asked to follow instructions (pointing to or moving tokens) of increasing length and syntactic complexity. Administration and norms according to Apt [31]

All tests have Swedish norms

*RG* reference group, *BeSS Bedömning av subtila språkstörningar*, assessment of subtle language disorders

All of the patients were also asked whether they had experienced any subjective change in their language, speech or communication during recent years. The responses were divided into three categories; *yes* (patients that clearly had experienced a change), *uncertain* (patients who were not sure but believed they had experienced some subtle changes, such as difficulty finding words) and *no* (patients who had not experienced any change). If the patient was uncertain, they were asked to elaborate upon the way in which they had experienced any change. If there was uncertainty about how to categorize a response, three of the authors (MA, LH, and FL) would discuss it until they achieved consensus.

### Data collection

All patients were tested by the first author before surgery (mean 14 days before surgery, range 1–72 days). The testing consisted of two sessions that lasted between 2 and 3 h with a longer break in between. It included a narrative writing task and a spontaneous speech task. These were both included in the project but were not analyzed in this study. All testing was video recorded to make it possible to double check when scoring was uncertain.

The reference group was tested and analyzed by five final-year students from the speech and language pathology programme at the University of Gothenburg.

Data concerning tumor characteristics were derived from patient records. Tumor localization was determined by a neurosurgeon (AJ) using T2-weighted/FLAIR images without knowledge of the language test results in individual patients. Location in language eloquent regions was categorized according to Chang et al. [20], and was further divided into three groups for the purpose of this study: (1) language eloquent areas in the left and presumed dominant hemisphere (LH), (2) non-language eloquent areas in the left and presumed dominant hemisphere and (3) the right presumed non-dominant hemisphere (RH).

### Statistical analysis

Differences between the patient group and reference group on the language tests were compared with Mann–Whitney U for independent samples or an independent t-test, depending on the data distribution. Additional statistical comparisons excluding the five patients with a glioma of a higher grade were also made. Since the study was exploratory, no adjustments were made for multiple comparisons. Statistical significance was set to p < .05.

The patients’ individual test scores were computed into z-scores using Swedish norms (see Table 2 for more information and references), and this enabled us to gain an overview of each individual’s performance. When applicable, the computation took age and/or educational level into consideration.

*IBM SPSS Statistics version 20* was used for computation.

### Ethical considerations

The study was approved by the regional ethical review board in Gothenburg. Written, informed consent was obtained from all participants.

## Results

### Results of language tests in patients with presumed LGG and in the reference group

Table 3 displays the results of the language tests conducted on the presumed LGG group and the reference group. The presumed LGG group performed significantly worse on the confrontation naming test BNT and on the word fluency category Animals. There was also a significant difference between the presumed LGG group and the reference group on the BeSS subtest *Comprehension of metaphors*. However, in this case the patients performed better than the participants in the reference group.

**Table 3:** Comparisons between patients with presumed LGG and a reference group on a set of language tests

Tests (max score)	LGG-group *N* = 23^a^		R-group *N* = 80		Sig.
	Mean SD	Mdn (min–max)	Mean SD	Mdn (min–max)	
BeSS total (210)	181.2 18.1	186 (144–205)	180.6 16.27	183.5 (137–200)	*U* = 840 *p* = .745
Subtests in BeSS (30)					
1. BeSS RLS	20.9 5.33	22 (8–27)	21.8 4.99	23 (8–30)	*U* = 802 *p* = .524
2. BeSS RS	26.0 3.91	26.5 (15–30)	25.3 3.41	26 (16–30)	*U* = *745.5* *p* = .267
3. BeSS MI	27.9 2.56	28 (22–30)	27.4 2.52	28 (21–30)	*U* = *749.5* *p* = .273
4. BeSS CLS	26.3 3.20	27 (21–30)	27.3 3.52	27.5 (12–30)	*U* = *701* *p* = .122
5. BeSS CA	25.1 4.96	26.5 (15–30)	25.9 4.31	27 (10–30)	*U* = 811 *p* = .567
6. BeSS CM	28.3 2.15	29 (23–30)	26.6 3.27	28 (16–30)	*U* = *585.5* *p* = .014*
7. BeSS WD	26.6 4.24	27.5 (15–30)	26.5 2.89	27 (14–30)	*U* = *737.5* *p* = .239
Sentence analysis (54)	50.2 6.74	52 (25–54)	50.6 4.98	52 (30–54)	*U* = *854* *p* = .827
Morphological completion (45)	42.1 4.08	42 (36–48)	42.5 4.73	45 (30–48)	*U* = *789.5* *p* = .451
BNT (60)	50.9 5.24	53 (37–58)	53.9 3.62	54 (41–59)	*U* = *653* *p* = .034*
FAS	43.0 12.7	43 (24–71)	45.5 10.6	46 (19–67)	*t* = − *0.948* *p* = .345
Animals	22.4 5.33	22 (13–32)	25.4 5.31	25 (8–41)	*t* = *− 2.098* *p* = .038*
Verbs	19.0 6.00	20 (6–30)	21.4 6.33	21 (10–40)	*t* = *− 1.860* *p* = .066

*R-group* reference group, *RLS* repetition of long sentences, *RS* recreating sentences, *MI* making inferences, *CL* comprehension of logico-grammatical sentences, *CA* comprehension of ambiguous sentences, *CM* comprehension of metaphors, *WD* word definitions, *SA* sentence analysis, *MC* morphological completion

^a^With exception for BeSS, n = 22

*Significant at level < 0.05. *U*-value reported for Mann–Whitney U test for independent samples, and *t*-value reported for student’s t-test

After additional analyses excluding the five patients who surprisingly after surgery were diagnosed with a high-grade glioma (HGG), Animals were no longer significantly different (LGG-group *mean* = 22.4, *SD* = 5.38, Reference group *mean* = 25.4, *SD* = 6.33, *t*(96) = − 1.863), *p* = .065). The results were not significantly altered in any other way after exclusion of the five HGG patients, thus we included the entire cohort of presumed LGG in the further analyses. Table A in the supplementary material displays all comparisons between the patients with confirmed LGG and the reference group.

### Language impairment in patients with presumed LGG related to localization in language eloquent area

The patients’ score on each test was compared to Swedish norms that are used in clinical practice (Table 2). A total of seven of the 23 patients (30.4%) performed below the cut-off level on at least one test or subtest. The corresponding percentage for the reference group was 27.5%.

Patients who performed below the cut-off level were found in each of the three tumor localization groups (Table 4). When the tumor was situated in language eloquent area, 2/7 performed below cut-off on at least one test or subtest. In the group with a left-sided tumor in a non-language eloquent area 2/9 performed below cut-off on at least one test or subtest. The figure was 3/7 in those with right-sided tumor.

**Table 4:** The patients’ results on all language tests, and report of subjective change in language or communication ability, divided by tumor eloquence

Tumor location	ID	Performance on language tests reported in z-scores (A-ning and Token test are reported in raw scores)																Subjective change in language or communication
		A-ning	BeSS	1.BeSS RLS	2. BeSS RS	3. BeSS MI	4. BeSS CL	5. BeSS CA	6. BeSS CM	7. BeSS WD	SA	MC	BNT	FAS	Animals	Verbs	Token test	
Language eloquent area in left hemisphere	**P4**	4.9	− 1.64	− 0.10	− 0.83	0.33	− 0.65	− **2.19**	− 0.42	**− 2.97**	0.44	− 0.45	− 0.25	0.67	− 1.55	**− 2.55**	33	Uncertain
	P12**	5.0	1.28	0.71	1.39	1.07	0.69	1.00	1.09	1.23	0.66	0.27	0.39	1.65	− 0.45	− 0.98	36	No
	P14**	5.0	0.47	0.51	1.39	1.07	− 1.95	0.36	1.09	− 0.06	0.66	− 1.73	0.12	− 1.8	0.17	0.11	36	No
	**P15**	5.0	0.99	1.12	0.83	0.33	− 0.52	1.00	1.09	1.23	− 0.59	0.27	− 0.42	− 1.88	− 1.86	− 0.36	36	No
	P25**	4.8	− 1.97	**− 2.76**	0.28	− 0.78	− 1.38	− 1.13	− 0.12	**− 3.61**	− 1.78	− 1.30	**− 2.45**	0.98	**− 2.04**	**− 2.06**	34	Uncertain
	**P32**	4.9	0.48	− 1.53	0.83	1.07	− 0.52	0.15	1.09	− 0.06	0.44	− 0.66	− 0.15	0.07	− 1.87	− 0.52	36	No
	**P36**	5.0	1.34	− 0.31	1.39	− 0.04	0.69	1.00	1.09	0.26	0.44	0.62	0.56	0.17	1.75	1.89	35	No
Non-language eloquent area in left hemisphere	P1**	5.0	0.62	0.71	1.39	− 1.52	− 1.95	1.00	− 0.73	0.26	0.07	0.62	1.04	− 0.75	− 1.23	0.40	36	No
	**P2**	5.0	0.94	0.71	0.28	1.07	− 0.34	0.57	1.09	1.23	0.03	0.93	0.26	0.3	0.35	0.27	36	No
	**P5**	5.0	0.82	0.92	0.83	1.07	0.90	− 0.49	1.09	1.23	0.66	− 1.07	− 1.37	− 1.65	− 0.76	0.11	36	Uncertain
	**P18**	5.0	1.08	0.92	− 0.56	− 0.04	− 1.95	1.00	1.09	1.23	0.81	− 1.30	− 0.87	− 0.23	− 1.08	− 1.30	36	No
	**P19**	4.9	0.36	− 0.31	0.28	0.33	0.69	0.57	1.09	0.26	0.66	− 0.40	− 0.29	− 1.43	− 1.25	− 0.16	35	No
	**P21**	5.0	0.09	0.31	− 1.94	1.07	0.00	− 0.06	0.18	0.58	0.07	− 1.30	0.09	0.76	0.17	0.19	**30**	No
	**P27**	4.9	1.38	0.92	0.56	0.33	0.90	1.00	1.09	1.23	0.44	1.26	− 0.07	1.27	− 1.08	1.47	35	Yes
	**P29**	4.9	0.65	0.10	1.39	1.07	0.69	0.36	0.48	0.26	0.66	− 0.40	− 0.96	− 0.63	− 0.86	− 0.16	35	Uncertain
	**P34**	4.9	− 1.38	− 1.94	**− 2.78**	1.07	− 0.65	− 0.06	0.48	0.58	0.66	0.27	− 0.42	− 1.64	0.95	0.11	33	No
Right hemisphere	P7**	5.0	0.76	0.71	0.83	0.33	− 0.52	1.00	1.09	0.58	0.66	− 0.84	− 0.29	− 0.53	0.17	1.20	36	No
	**P10**	4.9	− 0.38	− 0.71	0.00	1.07	− 0.52	**− 2.19**	0.48	− 0.39	0.26	− 0.02	− 0.23	− 0.03	− 0.45	− 1.09	35	No
	**P11**	5.0	–	–	–	–	–	–	–	–	–	–	0.88	1.76	1.11	0.62	35	No
	**P13**	5.0	0.01	0.51	0.00	1.07	− 0.34	− 0.91	0.48	0.58	0.66	0.27	− 0.96	0	0.48	0.73	35	No
	**P20**	5.0	− 0.38	− 1.53	− 0.28	− 1.52	0.90	− 1.34	0.48	1.23	0.07	1.26	0.72	− 0.51	0.02	− 0.02	36	No
	**P26**	4.9	− 1.14	− 0.51	0.83	− 0.04	**− 3.38**	− 1.13	0.48	**− 2.32**	**− 3.09**	0.27	− 0.01	− 1.33	− 1.31	− 0.36	35	No
	**P35**	4.8	− 1.11	− 1.33	− 0.28	− 1.89	− 1.95	− 0.70	− 1.03	0.26	**− 4.56**	− 0.66	0.4	0.92	− 0.92	− 1.72	**32**	Yes

The bolded scores are below the chosen cut-off and indicate a subnormal performance. For the z-transformed tests a cut-off of − 2 or was chosen. Token test has a cut-off at 33, which means that scores below 33 indicate difficulties in language comprehension. The A-ning score should be interpreted as follows: Mild = mean 4.5. Moderate/moderately severe = mean 3.2/3.4, Severe = mean 1.8, Very severe = mean 0.5 [21]

*RLS* repetition of long sentences, *RS* recreating sentences, *MI* making inferences, *CL* comprehension of logico-grammatical sentences, *CA* comprehension of ambiguous sentences, *CM* comprehension of metaphors, *WD* word definitions, *SA* sentence analysis, *MC* morphological completion.

**Patients revealed with a HGG after surgery

### Subjective change in language, speech or communicative ability prior to tumor treatment

Only two of the 23 patients (8.7%) described experiencing a clear change in their language ability (Table 4). Patient 27 reported problems with word retrieval that affected speech fluency, while patient 35 experienced word retrieval and spelling difficulties. Only patient 35 performed below cut-off level on a language test. These subjective changes had occurred anywhere from a few months up to a year prior to diagnosis.

The responses we received were sometimes ambiguous. For example, we categorized four of the patients (17.4%) as uncertain because they reported no clear experience of change in their language ability. However, some of them in fact reported word retrieval difficulties and problems remembering names. Some of the patients were in their fifties and sixties and simply related their problems to the normal ageing process. Two of the patients in this group performed below cut-off level on at least one language test.

Seventeen patients (73.9%) had experienced no change. Four of them (patients 10, 21, 26 and 34) scored below normal on at least one test.

The last column in Table 4 contains information the patients gave about subjective changes in their *language* or *communicative ability*. Four patients reported experiencing transient *motor* speech difficulties (patients 7, 12, 25, and 32). Three of these had experienced periods of slurred speech but one had only noticed this when they spoke English. The fourth patient (no. 25) had experienced twitching in his mouth in the last year and found that once or twice a day, he got stuck trying to enunciate a word, almost as though he was stammering. This had begun following an epileptic seizure a few months earlier and had persisted since then. Furthermore, this patient turned out to have a glioma of grade III. All patients with HGG are marked in Table 4 with asterisks.

## Discussion

Overall, the patients with presumed LGG performed worse on tests of lexical retrieval (naming and semantic word fluency) than did the participants of a reference group that was matched for age and educational level. Still, most of the patients had normal or nearly normal language ability and the number of scores below the normal range was only slightly higher in the presumed LGG group than it was in the reference group. Only a few patients reported subjective language deficits. The findings of lexical retrieval problems concur with those of earlier research [8, 9, 15, 16]. Although the presumed LGG group performed worse than the reference group in these tests, only two patients performed below the cut-off level of − 2 z-scores.

After surgery, five of the patients turned out to have a glioma of a higher grade. After additional analyses excluding these patients, the results were similar except for the difference in one of the semantic word fluency measures that no longer differed between the groups. Four of the five patients performed within the normal range and had not experienced any change in their language or communication. However, one patient performed below the normal range on several tests. If his performance was related to tumor grade is not clear.

Overall, the language impairments seen in this study were minor. Only a few patients performed below normal on several of the tests conducted and none of them tested positive for mild aphasia on the A-ning test. This may support the findings of earlier research that suggest that aphasia test batteries are not sensitive enough to detect subtle changes in language ability [8, 32]. We therefore included other, more sensitive tests as well (lexical retrieval and a test of HLL; BeSS). HLL is an overarching term for more complex language abilities that require extensive language and cognitive processing [18]. The BeSS test was chosen since both language and cognitive deficits may be found in patients with LGG and researchers have noted the need for assessments of subtle language impairment [8, 32].

In our study the only differences in BeSS found between the two groups were on the subtest ‘Comprehension of metaphors’. Surprisingly, the presumed LGG group as a whole scored significantly better than the reference group. Whether this is a true difference, a significant difference due to chance, or an inherent difficulty in measuring comprehension of metaphors is not clear. Six patients scored less than − 2 z-scores on at least one subtest in the BeSS. Studies of larger samples and including results from post-surgical assessment are needed in order to see whether BeSS is sufficiently sensitive to detect language impairment in this patient population.

Only a slightly higher proportion of the presumed LGG group (30.4%) performed below cut-off on at least one test or subtest than the reference group (27.5%). To make it possible to compare these results to those of other studies, it may be better to look specifically at the number of patients with a tumor in the LH who performed subnormally on a language test. Four of 16 patients (25%) with a tumor in the LH performed below the cut-off level on one test or subtest and only two patients (12.5%) had scores below the cut-off level on several tests. These figures seem comparable to those of other studies that have found mild language impairment to be present in 10.4–36.4% of patients [4–6, 10]. It has been suggested that changes in language ability may be mild even when the tumor is situated in language eloquent area because neural language networks may have time to become reorganized with slow-growing brain tumors [2, 33].

In our small study including only a few patients with subnormal language function we could not demonstrate the importance of tumor location for language impairment. In a larger sample of patients with gliomas in language eloquent cortex and non-language eloquent areas of the LH, Satoer et al. [13] also found that the location of the tumor did not appear to be relevant. In our study, we grouped the patients according to the proximity of their tumor to the language eloquent area. Of the patients that performed at a subnormal level on three or more tests, two had a tumor in language eloquent cortex in LH and one had a tumor in the RH. It is debateable as to what actually constitutes a language eloquent area. We categorized the tumors according to anatomical landmarks rather than on functional imaging [20], where the latter may provide more accurate information about the individual’s language areas.

It could be argued that the patient’s experience of language impairment is the most important issue, but this is often overlooked. In our study, few of the patients had noticed any change in their language or communicative ability. The two who reported a change said they had had problems finding words. However, several of the patients who were uncertain as to whether they had experienced any changes also said they had problems finding words sometimes. Satoer et al. [9] found that as many as 56.5% of the patients they studied had experienced problems finding words before surgery, despite showing normal language ability or a minimal handicap on the Aphasia Severity Rating Scale [34]. This inconsistency suggests that word-finding problems may be difficult to detect using existing standard language tasks.

Some patients reported that they had experienced changes in their motor speech ability. Motor speech deficits are not commonly reported in this patient group. However, seizures may result in these symptoms. Duffau et al. [35] reported that of 25 patients who had a tumor in or near the prefrontal cortex, eight had experienced partial seizures with transient speech disturbances. Similar findings have been reported in patients who have a tumor in the opercular region [36]. All of the patients in our study who reported speech disturbances had had seizures, but only one of them related the speech disturbances to the seizures. However, it is possible that his motor speech impairment and/or persistent seizures caused reduced performance on the language tasks.

We found no clear pattern in the relationship between subjectively experienced language problems and objective test results, which at least partly could be attributed to the low number of patients demonstrating a language impairment. As Satoer et al. [9] also observed, some of the patients who report problems finding words perform normally in language tests. A similar discrepancy between self-evaluation and formal testing was also found by Pahlsson et al. [37], who investigated motor and cognitive disability in patients with LGG. The discrepancy between objective and subjective evaluations may reflect the fact that subtle changes in language or other cognitive abilities may be difficult to identify. Some of the patients in our study achieved the maximum score on several tests and subtests and this made it difficult to know whether there may nonetheless be subtle changes. In patients who had a high level of language functioning prior to the development of their tumor, it may be particularly difficult to detect subtle signs of deterioration. It is therefore important to consider self-reported problems and not rely solely on a test score.

Tests of lexical retrieval, such as naming and word fluency, seem to be sensitive methods of measuring language impairment in patients with LGG. This is borne out by the fact that we observed differences between the presumed LGG group and the reference group. The standard aphasia test A-ning did not identify language impairment in any patient, whereas most of the other tests identified impairment in at least one patient. Since testing patients before surgery provides a baseline against which to compare any changes in language ability following tumor treatment, a post-operative evaluation of the test battery is needed. Firstly, to fully investigate what tests are best suitable for this patient population, and secondly to identify which patients who benefit from an extensive testing and which are better suited for a shorter screening. Furthermore, an investigation if and how subtle language disorders affect these patients is needed.

## Conclusion

Most patients in this study with presumed LGG had normal or nearly normal language function prior to surgical treatment. We therefore recommend conducting a thorough assessment of their language ability before surgery to identify possible subtle language deficits and to establish a baseline against which to compare language functioning at post-treatment follow-ups.

## Electronic supplementary material

Below is the link to the electronic supplementary material.

Supplementary material 1 (DOCX 16 KB)
