# Supplementary material for: Pre-operative language ability in patients with presumed low-grade glioma
Source: J Neurooncol. 2017 Dec 1;137(1):93–102. doi: 10.1007/s11060-017-2699-y (PMC5846960; doi:10.1007/s11060-017-2699-y)
Supplement: Supplementary file 1 — Supplementary material 1 (DOCX 16 KB) [file 11060_2017_2699_MOESM1_ESM.docx]

**Supplementary material**

**Table A** Comparisons between patients with confirmed LGG and a reference group on a set of language tests

| Tests (max score) | **LGG-group**  *N* = 18^1^ | |  | **R-group**  *N* = 80 | |  |  |
| --- | --- | --- | --- | --- | --- | --- | --- |
|  | Mean  SD | Mdn  (min-max) |  | Mean  SD | Mdn  (min-max) |  | Sig. |
| **BeSS total** (210)  Subtests in BeSS (30): | 180.4  17.0 | 183  (149-201) |  | 180.6  16.27 | 183.5  (137-200) |  | *U =* 679  *p* = .992 |
| 1. BeSS RLS | 20.7  4.81 | 22  (12-27) |  | 21.8  4.99 | 23  (8-30) |  | *U =* 583  *p* = .356 |
| 2. BeSS RS | 25.1  3.40 | 26.5  (15-30) |  | 25.3  3.41 | 26  (13-30) |  | *U =* 679  *p* = .992 |
| 3. BeSS MI | 28.1  2.45 | 28  (22-30) |  | 27.4  2.52 | 28  (21-30) |  | *U* = 549  *p* = .199 |
| 4. BeSS CLS | 26.5  3.39 | 27  (21-30) |  | 27.3  3.52 | 27.5  (12-30) |  | *U* = 569  *p* = .261 |
| 5. BeSS CA | 24.4  5.04 | 26.5  (15-30) |  | 25.9  4.31 | 27  (10-30) |  | *U =* 561  *p* = .251 |
| 6. BeSS CM | 28.4  2.00 | 29  (23-30) |  | 26.6  3.27 | 28  (16-30) |  | *U* = 439  *p* = .**019*** |
| 7. BeSS WD | 27.0  3.76 | 27.5  (17-30) |  | 26.5  2.89 | 27  (14-30) |  | *U* = 539  *p* = .174 |
| **Sentence analysis** (54) | 50.1  7.10 | 52  (25-54) |  | 50.6  4.98 | 52  (30-54) |  | *U* = 674.5  *p* = .957 |
| **Morphological completion** (45) | 42.6  3.95 | 42  (36-48) |  | 42.5  4.73 | 45  (30-48) |  | *U* = 650.5  *p* = .774 |
| **BNT** (60) | 51.1  4.18 | 53  (42-57) |  | 53.9  3.62 | 54  (41-59) |  | *U* = 436  *p* = **.009*** |
| **FAS** | 43.6  12.4 | 43  (24-71) |  | 45.5  10.6 | 46  (19-67) |  | *t* = -.644  *p* = .521 |
| **Animals** | 22.4  5.38 | 22  (13-32) |  | 25.4  5.31 | 25  (8-41) |  | *t* = -1.863  *p* = .065 |
| **Verbs** | 18.8  5.76 | 20  (6-27) |  | 21.4  6.33 | 21  (10-40) |  | *t* = -1.838  *p* = .069 |

Abbreviations: R-group = reference group, RLS = Repetition of long sentences, RS = Recreating sentences, MI = Making inferences, CL = Comprehension of logico-grammatical sentences, CA = Comprehension of ambiguous sentences, CM = Comprehension of metaphors, WD = Word definitions, SA = Sentence analysis, MC = Morphological completion.

Notes: ^1^With exception for BeSS, n=17. * Significant at level < .05. *U*-value reported for Mann-Whitney U test for independent samples, and *t*-value reported for student's t-test.
